# Impella 5.5 Support in Severe Cardiogenic Shock: Outcomes From the First Spanish Multicentre Cohort in Heart Transplant Centres

**DOI:** 10.1093/icvts/ivag137

**Published:** 2026-05-13

**Authors:** José Manuel Martínez-Comendador, Elio Martín Gutiérrez, Daniel Ortiz Berbel, Lucía García Alcalde, Álvaro Pedraz Prieto, Christian Muñoz-Guijosa, Carlos Esteban Martín López, Stefano Urso, Gregorio Rábago Juan Aracil, José Joaquín Cuenca Castillo, Manuel Pérez-Guillén

**Affiliations:** Department of Cardiac Surgery, A Coruña University Hospital Complex (CHUAC), A Coruña, 15006, Spain; Biomedical Research Institute of A Coruña (INIBIC), A Coruña, 15006, Spain; Department of Cardiovascular Surgery, Virgen de la Arrixaca University Hospital, Murcia, 30120, Spain; Murcian Institute for Biosanitary Research (IMIB Arrixaca), Murcia, 30120, Spain; Department of Cardiac Surgery, Bellvitge University Hospital, L’Hospitalet de Llobregat, Barcelona, 08907, Spain; Department of Cardiac Surgery, Marqués de Valdecilla University Hospital, Santander, Cantabria, 39008, Spain; Department of Cardiac Surgery, Gregorio Marañón University General Hospital, Madrid, 28007, Spain; Department of Cardiac Surgery, 12 de Octubre University Hospital, Madrid, 28041, Spain; Department of Cardiac Surgery, Puerta de Hierro–Majadahonda University Hospital, Majadahonda, Madrid, 28222, Spain; Department of Cardiac Surgery, Dr. Negrín University Hospital of Gran Canaria, Las Palmas de Gran Canaria, 35010, Spain; Department of Cardiac Surgery, Clínica Universidad de Navarra, Pamplona, 31008, Spain; Department of Cardiac Surgery, A Coruña University Hospital Complex (CHUAC), A Coruña, 15006, Spain; Biomedical Research Institute of A Coruña (INIBIC), A Coruña, 15006, Spain; Department of Cardiac Surgery, La Fe University and Polytechnic Hospital, Valencia, 46026, Spain

**Keywords:** cardiogenic shock, ventricular assist device, Impella 5.5, heart transplantation, temporary mechanical circulatory support

## Abstract

**Objectives:**

Impella 5.5 is an established temporary mechanical circulatory support (tMCS) device for severe cardiogenic shock. Spain, characterized by high heart transplant activity and increasing use of controlled donation after circulatory death (DCD), provides a relevant setting to evaluate its use and outcomes. We aimed to describe clinical characteristics, trajectories, and outcomes of Impella 5.5 support in the first Spanish multicentre cohort from heart transplant centres.

**Methods:**

Retrospective multicentre observational study including consecutive patients supported with Impella 5.5 in hospitals with active heart transplant programmes. Baseline characteristics, Interagency Registry for Mechanically Assisted Circulatory Support (INTERMACS) profile, additional support, complications, support duration, and in-hospital mortality were recorded. Outcomes were analysed across INTERMACS profiles.

**Results:**

Ninety-three patients were included (33 INTERMACS 1, 37 INTERMACS 2, 23 INTERMACS 3). Additional support was required in 46%, mainly venoarterial extracorporeal life support (VA-ECLS). Device-related complications were reported as event frequencies. The predominant clinical trajectory was heart transplantation (68.8%), contrasting with international series in which long-term mechanical support and transplantation are more evenly distributed. In-hospital mortality was 43%, with a clear gradient across INTERMACS profiles 1-3 (60.6%, 37.8%, and 26.1%, respectively; *P* = .013). Mean support duration was 16 days, and more than one-third of transplants were from controlled DCD donors.

**Conclusions:**

Impella 5.5 was used as tMCS in severe cardiogenic shock and was predominantly associated with heart transplantation in a transplant-oriented healthcare system. Outcomes were closely associated with INTERMACS profile, reflecting differences in baseline severity and patient selection. Comparisons with international registries are descriptive only.

## Graphical Abstract

**Figure:**
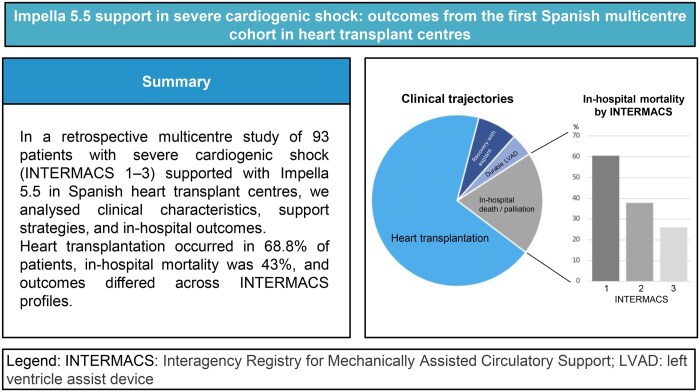

## INTRODUCTION

Cardiogenic shock remains one of the highest-mortality scenarios in cardiology and cardiac surgery. Despite advances in pharmacological therapy and percutaneous interventions, many patients require temporary mechanical circulatory support (tMCS) to maintain forward flow, limit end-organ injury, and enable timely decision-making.

The Impella 5.5 (Johnson & Johnson MedTech, Villmergen, Switzerland) is a surgically implanted microaxial left ventricular assist device (LVAD) capable of delivering more than 5 L/min of forward flow, with low haemolysis rates and durability that may exceed 1 month. Implantation through the axillary artery enables prolonged support and early mobilization, distinguishing it from other tMCS modalities such as venoarterial extracorporeal membrane oxygenation (VA-ECLS), which is associated with a higher burden of haematologic and vascular complications, and from extracorporeal ventricular assist devices requiring more invasive surgical approaches.

Evidence on Impella 5.5 initially emerged from single-centre experiences^1–5^ and has expanded in recent years through large multicentre registries involving patients with cardiogenic shock.^6–9^ These studies describe its use as a bridge to recovery, heart transplantation, or durable LVAD implantation. However, the applicability of these findings may vary across healthcare systems with different organizational structures, transplant activity, and access to donor organs. In Spain, despite high heart transplant activity and an increasing controlled donation after circulatory death (DCD) programme, national registry data do not differentiate between specific tMCS devices, limiting technology-specific interpretation.^10^ Moreover, the Spanish transplant system, coordinated nationally through the Spanish National Transplant Organization (ONT), provides a donor availability context that differs from many other European healthcare settings and may shape the final trajectory of patients supported with tMCS.

Despite the progressive adoption of Impella 5.5 in Spanish heart transplant centres, no national multicentre series specifically focused on this device has been reported to date. We therefore aimed to describe the clinical characteristics, Interagency Registry for Mechanically Assisted Circulatory Support (INTERMACS) profile, clinical trajectories, and in-hospital outcomes of patients supported with Impella 5.5 in Spanish heart transplant centres, and to contextualize these results within the framework of the national healthcare system and contemporary international multicentre registries.

## METHODS

### Patients and procedures

We conducted a retrospective multicentre observational study including all consecutive cases of Impella 5.5 implantation performed in Spanish heart transplant centres from the first recorded case in January 2023 to the most recent implantation on September 1, 2025, with no exclusions. All hospitals with an active heart transplant programme that had implanted at least one Impella 5.5 device during the study period were included.

All patients in whom Impella 5.5 was used as tMCS were eligible, irrespective of the initial indication, including ischaemic cardiogenic shock, acute decompensation of advanced heart failure, postcardiotomy shock, or ventricular dysfunction of other aetiologies associated with refractory shock. Decisions regarding device implantation were made according to routine clinical practice at each participating centre, in accordance with the principles outlined in international consensus documents on tMCS.

Patients were retrospectively stratified according to the INTERMACS classification, which serves as an international reference framework for advanced heart failure. In this cohort, only INTERMACS profiles 1 to 3 were recorded, corresponding to the most severe clinical presentations.

Clinical data were collected from medical records/local databases and included comorbidities; haemodynamics at implantation (SBP/MAP, serum lactate); renal function; prior cardiac arrest (CA) or intubation; shock aetiology; mechanical support strategy; implantation approach; support duration; and in-hospital outcomes.

#### Definitions of clinical trajectories 

Clinical trajectories after Impella 5.5 implantation were defined by the main in-hospital outcome: heart transplantation, durable LVAD implantation, recovery with device explant, or death/palliation.

Major complications were recorded according to criteria used in international series. Clinical trajectories followed the terminology proposed in the 2025 tMCS consensus. Only definitive in-hospital outcomes were considered: heart transplantation, durable LVAD implantation, recovery with device explantation, or death/palliation. In-hospital mortality included all deaths during support or after withdrawal of support, including cases redirected towards palliation. Thirty-day mortality was not a predefined end-point; outcomes are therefore reported as in-hospital mortality.

### Ethical statement

This registry was approved by the appropriate Biomedical Research Ethics Committee, which authorized the use of anonymized clinical data. Owing to its retrospective and observational nature, individual informed consent was waived. No biological samples were collected, and no biobank or database for future unspecified research use was created.

### Statistical analysis

Normality of continuous variables was assessed using the Kolmogorov-Smirnov test. Data are presented as mean ± standard deviation. Comparisons across INTERMACS profiles were performed using analysis of variance (ANOVA) for normally distributed variables or the Kruskal-Wallis test for non-normally distributed variables. Categorical variables are expressed as percentages and were compared using the χ^2^ test or Fisher’s exact test, as appropriate. A 2-sided significance level of α = 0.05 was considered statistically significant.

## RESULTS

### Baseline characteristics

The cohort comprised 93 patients supported with Impella 5.5, classified as INTERMACS 1 (*n* = 33), INTERMACS 2 (*n* = 37), and INTERMACS 3 (*n* = 23). Mean age was 54.7 ± 12.4 years, and 9.7% of patients were women. Arterial hypertension (39.8%) and dyslipidaemia (48.4%) were the most frequent comorbidities, with no relevant differences across INTERMACS profiles. Baseline characteristics are summarized in **Table 1**.

**Table 1.** Pre-Implant Characteristics and Shock Profile

Pre-implant characteristics					
	Overall (*n* = 93)	INTERMACS 1 (*n* = 33)	INTERMACS 2 (*n* = 37)	INTERMACS 3 (*n* = 23)	*P*-value
Age, years	54.7 ± 12.4	55.6 ± 11.7	53.6 ± 13.2	54.1 ± 14.5	.476
Female sex	9.7% (9)	9.1% (3)	5.4% (2)	17.4% (4)	.309
Hypertension	39.8% (39)	27.3% (9)	54.1% (20)	34.8% (8)	.063
Dyslipidaemia	48.4% (45)	54.5% (18)	45.9% (17)	43.5% (10)	.667
Diabetes mellitus	29.1% (27)	18.2% (6)	37.8% (14)	30.4% (7)	.192
Smoking history	62.3% (58)	72.7% (24)	51.4% (19)	65.2% (15)	.174
Prior myocardial infarction	44.1% (41)	57.6% (19)	37.8% (14)	34.8% (8)	.147
Prior percutaneous coronary intervention	43.1% (40)	54.5% (18)	37.8% (14)	34.8% (8)	.243
KDIGO stage	1.97 ± 0.9	1.88 ± 0.9	2.16 ± 0.9	1.78 ± 0.7	.233
Creatinine clearance (mL/min)	75.0 ± 32.7	74.6 ± 26.1	68.1 ± 31.3	86.1 ± 39.6	.144

Shock characterization at implantation					
	Overall (*n* = 93)	INTERMACS 1 (*n* = 33)	INTERMACS 2 (*n* = 37)	INTERMACS 3 (*n* = 23)	*P*-value
Systolic blood pressure, mmHg	88.4 ± 14.6	84.6 ± 18.4	87.4 ± 12.9	95.2 ± 7.8	.033
Mean arterial pressure, mmHg	64.8 ± 11.6	63.8 ± 14.3	65.5 ± 9.8	65.0 ± 10.9	.856
Serum lactate, mmol/L	2.8 ± 2.8	6.1 ± 4.4	2.7 ± 2.2	2.0 ± 0.0	.195
Prior cardiac arrest	17.2% (16)	39.4% (13)	5.4% (2)	4.3% (1)	<.0001
Prior orotracheal intubation	42.0% (39)	78.8% (26)	29.7% (11)	8.7% (2)	<.0001
AKI	6.7% (6)	12.1% (4)	5.4% (2)	0% (0)	.764
Aetiology of shock					
Acute ischaemic	50.5% (47)	78.8% (26)	40.5% (15)	26.1% (6)	<.0001
Acute non-ischaemic	49.5% (46)	21.2% (7)	59.5% (22)	73.9% (17)	
Anterior myocardial infarction	48.4% (45)	69.7% (23)	43.2% (16)	26.1% (6)	<.0001
Coronary artery disease					
Number of diseased vessels	1.12 ± 1.4	1.7 ± 1.2	1.1 ± 1.5	0.6 ± 0.9	.003
Left main coronary artery disease	18.3% (18)	39.4% (13)	10.8% (4)	0% (0)	<.0001

The mean number of diseased vessels was calculated only among patients with documented coronary artery disease.

Abbreviations: AKI, acute kidney Injury; INTERMACS, Interagency Registry for Mechanically Assisted Circulatory Support; KDIGO, Kidney Disease: Improving Global Outcomes.

The haemodynamic profile reflected greater severity among INTERMACS 1 patients, who exhibited lower systolic blood pressure and higher serum lactate levels. Prior CA (39.4%) and endotracheal intubation (78.8%) were significantly more frequent in this group. Acute ischaemic aetiology predominated among INTERMACS 1 patients (78.8%), whereas non-ischaemic causes were more common in INTERMACS 2 and 3 profiles. Among patients with non-MI-related cardiogenic shock, the main aetiologies were idiopathic/non-ischaemic dilated cardiomyopathy (*n* = 21), ischaemic dilated cardiomyopathy (*n* = 8), dilated-phase obstructive hypertrophic cardiomyopathy (*n* = 4), myocarditis (*n* = 3), refractory electrical storm (*n* = 3), post-transplant graft dysfunction/failure (*n* = 3), anthracycline-induced toxic cardiomyopathy (*n* = 2), post-cardiotomy shock (*n* = 1), and end-stage Fontan physiology (*n* = 1). Coronary artery disease burden was also greater in the most severe presentations, with a higher prevalence of multivessel disease and left main coronary artery (LMCA) involvement in INTERMACS 1 patients (**Table 1**).

### Type of mechanical circulatory support

**Figure 1.**
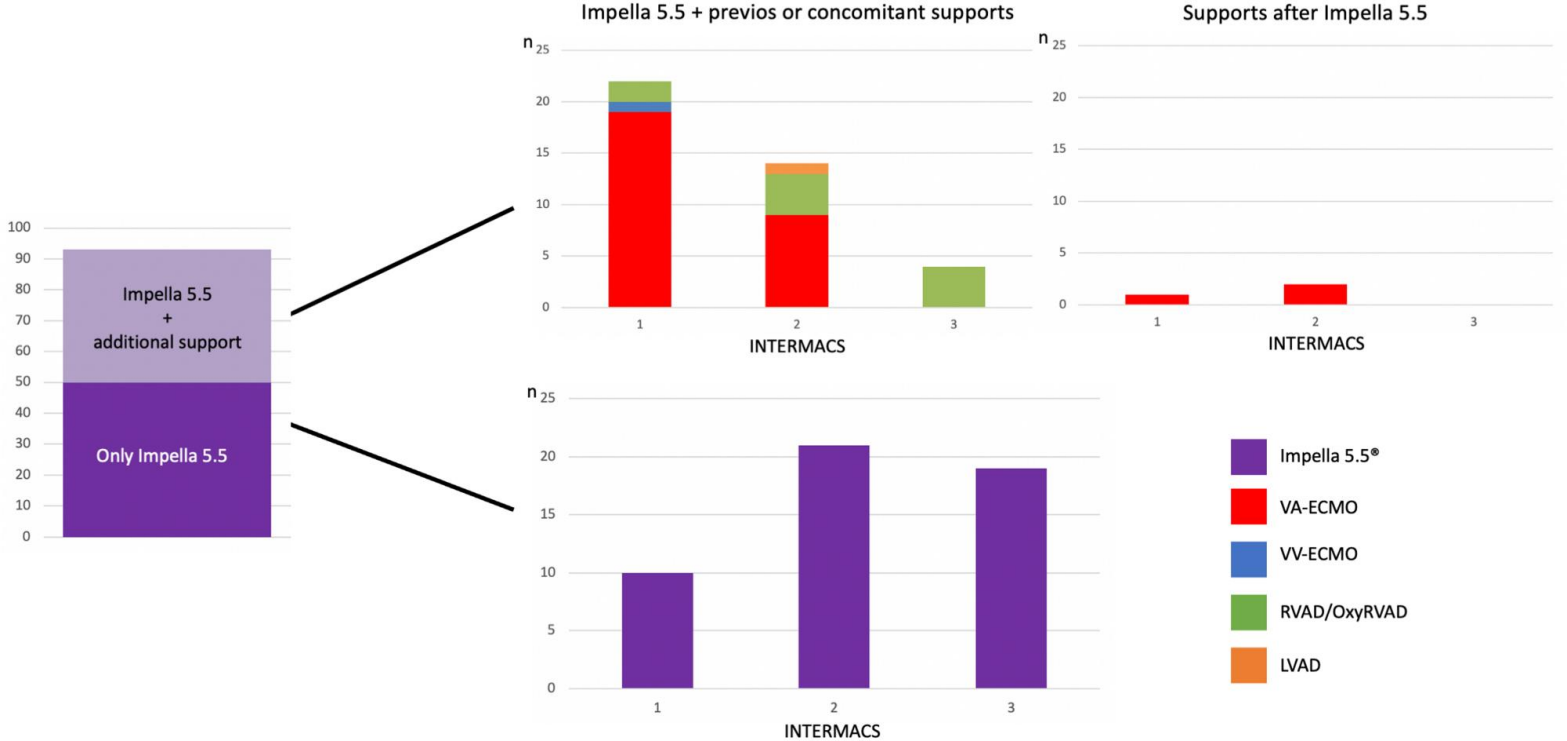
Distribution of Mechanical Circulatory Support Strategies by INTERMACS Profile. Abbreviations: INTERMACS, Interagency Registry for Mechanically Assisted Circulatory Support; LVAD, durable left ventricular assist device; OxyRVAD, right ventricular assist device with membrane oxygenator; RVAD, right ventricular assist device; VA-ECLS, venoarterial extracorporeal life support; VV-ECLS, venovenous extracorporeal life support

Overall, 53.8% of patients were managed exclusively with Impella 5.5, whereas 46.2% required additional tMCS. The most frequent additional support configuration involved venoarterial extracorporeal membrane oxygenation (VA-ECLS), accounting for 72% of cases receiving additional support and occurring predominantly among INTERMACS 1 patients (*P* < .0001). Among patients requiring sequential or combined tMCS, the most frequent support configurations involved VA-ECLS (*n* = 31) and RVAD/OxyRVAD (*n* = 10). Among patients exposed to VA-ECLS and Impella 5.5 (*n* = 31), Impella 5.5 was implanted first in 6 patients, whereas in the remaining 25, it was added after VA-ECLS initiation, mainly as a ventricular unloading and de-escalation strategy. The interval between the first and second device was 0.3 ± 2.3 days in the Impella-first subgroup and 3.4 ± 4.6 days in the VA-ECLS-first subgroup (*P* = .125). VA-ECLS weaning was achieved in 5 of 6 patients (83.3%) in the Impella-first subgroup and in 15 of 25 patients (60.0%) in the VA-ECLS-first subgroup. These findings are descriptive. Among patients exposed to Impella 5.5 and RVAD/OxyRVAD support (*n* = 10), right-sided support was added after Impella 5.5 initiation in all cases, at 5.4 ± 4.3 days; mortality in this subgroup was 40%. The axillary artery was the predominant implantation route (88.6%); these support strategies are summarized in **Table 2** and **Figure 1**.

**Table 2.** Mechanical Circulatory Support Strategy and Device-Related Morbidity

Mechanical circulatory support strategy					
	Overall (*n* = 93)	INTERMACS 1 (*n* = 33)	INTERMACS 2 (*n* = 37)	INTERMACS 3 (*n* = 23)	*P*-value
Only Impella 5.5	53.8% (50)	30.3% (10)	56.8% (21)	82.6% (19)	.001
Impella 5.5 + additional support	46.2% (43)	69.6% (23)	43.2% (16)	17.3% (4)	.001
VA-ECLS^a^	72.0% (31)	86.9% (20)	68.7% (11)	0% (0)	<.0001
VV-ECLS^a^	2.0% (1)	4.3% (1)	0% (0)	0% (0)	
RVAD/OxyRVAD^a^	23.2% (10)	8.6% (2)	25% (4)	100% (4)	
LVAD^a^	2.0% (1)	0% (0)	6.2% (1)	0% (0)	
Impella 5.5 implantation approach					
Axillary	88.6% (86)				
Central	7.2% (7)				
Ascending aorta	4				
Brachiocephalic trunk	3				

a Percentages refer only to patients with Impella 5.5 plus additional support.

**Table:** 

Impella 5.5-related morbidity	
	Overall (*n* = 93)
Bleeding	8.2% (8)
Surgical review	8.2% (8)
Device repositioning	16.5% (16)
Ischaemic stroke	6.2% (6)
Device thrombosis/dysfunction	8.2% (8)
Major haemorrhage	7.2% (7)
Right ventricular dysfunction	3.1% (3)

Mechanical circulatory support strategies and Impella 5.5-related morbidity.

Systematic laboratory assessment of haemolysis was not consistently available across participating centres and therefore haemolysis-related events could not be reliably analysed.

a Percentages refer only to patients receiving Impella 5.5 in combination with additional mechanical circulatory support.

Abbreviations: OxyRVAD, right ventricular assist device with membrane oxygenator; RVAD, right ventricular assist device; VA-ECLS, venoarterial extracorporeal life support; VV-ECLS, venovenous extracorporeal life support.

### Device-related complications

Complications attributable to Impella 5.5 were observed and are reported as event frequencies within this cohort. Local bleeding and the need for surgical revision occurred in 8.2% of the cases, and device repositioning was required in 16.5%. Acute kidney injury (AKI) requiring renal replacement therapy was observed in 6.7% of the patients, and ischaemic stroke occurred in 6.2%. Mechanical complications, including device thrombosis or dysfunction, and major bleeding events were observed at low absolute frequencies (**Table 2**).

### Clinical trajectories

Recovery sufficient to allow device explantation without subsequent advanced therapy was achieved in 7.5% of cases (7 out of 93 patients).

The predominant clinical trajectory was heart transplantation, which was performed in 64 patients, representing 68.8% of the overall cohort. This proportion was significantly higher among INTERMACS 2 (78.3%) and INTERMACS 3 (78.2%) patients (*P* = .016). More than one-third of transplanted patients received grafts from controlled DCD. For logistical reasons, organs from DCD were more frequently used in INTERMACS 1 patients (82.3%) than in INTERMACS 2 and 3 patients (20.6% and 22.2%, respectively; *P* = .003).

In contrast, only 4 patients (4.3% of the total cohort) underwent durable LVAD implantation as definitive therapy, a proportion markedly lower than that reported in international series. Among patients receiving concomitant VA-ECLS support, successful decannulation from extracorporeal support was achieved in 65.6% of cases. These outcomes are detailed in **Table 3**.

**Table 3.** Clinical Trajectories, Support Duration, and Outcomes Clinical Outcomes

	Overall (*n* = 93)	INTERMACS 1 (*n* = 33)	INTERMACS 2 (*n* = 37)	INTERMACS 3 (*n* = 23)	*P*-value
Bridge to					
Recovery	7.5% (7)	9.1% (3)	10.8% (4)	0% (0)	.001
Heart transplantation	68.8% (64)	51.5% (17)	78.3% (29)	78.2% (18)	
Durable LVAD implantation	4.3% (4)	6.0% (2)	0 (0%)	8.6% (2)	
Death/palliation (primary trajectory)	19.4% (18)	36.4% (12)	8.1% (3)	13.0% (3)	
Heart transplantation, organ source					
Donation after DCD^a^	37.5% (24)	82.3% (14)	20.6% (6)	22.2% (4)	.003^b^
Donation after brain death^a^	62.5% (40)	9.0% (3)	79.3% (23)	77.7% (14)	
ECLS weaning^a^	65.6% (21)	61.9% (13)	72.7% (8)	0% (0)	.437
In-hospital mortality	43.0% (40)	60.6% (20)	37.8% (14)	26.1% (6)	.013
Hospital discharge	57.0% (53)	39.4% (13)	62.2% (23)	73.9% (17)	
Duration of mechanical support (days)					
Impella 5.5	16.2 ± 14.9	17.8 ± 18.3	15.6 ± 11.9	15.0 ± 14.3	.794
ECLS	15.9 ± 12.8	17.5 ± 15.7	9.8 ± 5.7	n/a	.111
Total mechanical support	19.0 ± 16.0	22.4 ± 20.4	17.7 ± 11.7	16.3 ± 14.1	.301
Time from weaning of mechanical support to outcome (days)					
To in-hospital death	55.4 ± 114.8	49.3 ± 119.2	80.7 ± 131.1	18.0 ± 17.9	.515
Hospital discharge	40.4 ± 36.5	55.6 ± 50.3	38.2 ± 30.8	31.7 ± 29.1	.194

Clinical trajectories immediately following Impella 5.5 support, duration of mechanical circulatory support, and overall in-hospital outcomes in the study cohort.

a Percentages refer only to transplanted patients.

b *P-*value derived from comparison between INTERMACS 1 and INTERMACS 2-3 combined.

Abbreviations: DCD, donation after circulatory death; ECLS, extracorporeal life support; LVAD, durable left ventricular assist device.

### In-hospital survival

Overall in-hospital mortality was 43.0%, with a clear gradient according to INTERMACS severity: 60.6% in INTERMACS 1, 37.8% in INTERMACS 2, and 26.1% in INTERMACS 3 patients. Accordingly, the rate of hospital discharge increased progressively from INTERMACS 1 (39.4%) to INTERMACS 3 (73.9%) (see **Table 3**).

### Duration of support

Mean duration of Impella 5.5 support was 16.2 ± 14.9 days, and no statistically significant differences were detected across INTERMACS profiles. When VA-ECLS was used as an additional support device, its mean duration was 15.9 ± 12.8 days. Total duration of mechanical circulatory support, considering all devices combined, was 19.0 ± 16.0 days, with no statistically significant differences detected between groups.

### Additional analyses

Mortality was significantly higher among patients with prior CA (68.8%; *P* = .029) or endotracheal intubation before support initiation (69.2%; *P* < .0001). Patients managed exclusively with Impella 5.5 exhibited lower mortality than those requiring additional tMCS (30.0% vs 60.5%; *P* = .003). No statistically significant differences in survival were detected according to duration of Impella 5.5 support or total duration of mechanical circulatory support across all devices.

## DISCUSSION

This first multicentre Spanish Impella 5.5 cohort describes severity, trajectories, and in-hospital outcomes in heart transplant centres, adding context to international registries within a system with high transplant activity and increasing controlled DCD use.^6–9^

### Interpretation of the Spanish cohort

Patients in this series presented with marked clinical severity, with a high proportion of INTERMACS 1 and 2 profiles. The frequent occurrence of prior CA and endotracheal intubation among INTERMACS 1 patients reflects profound shock with early multiorgan dysfunction. Despite this, more than half of the cohort survived to hospital discharge. Outcomes were more favourable among INTERMACS 2 and 3 patients, whereas INTERMACS 1 patients experienced significantly higher mortality, consistent with the established association between shock severity at implantation and prognosis.

### Clinical trajectories and organizational context

The distribution of therapeutic trajectories in this cohort differs from that reported in international series. Whereas US and European registries describe a more balanced distribution between heart transplantation, durable LVAD implantation, and recovery,^6–8^ the predominant trajectory in the present series was heart transplantation, achieved in nearly 70% of patients.

This pattern likely reflects the organizational characteristics of the Spanish healthcare system, including high donor availability, a transplant-oriented structure, and prioritization of transplantation over long-term mechanical support. The extensive use of controlled DCD, particularly among INTERMACS 1 patients, further illustrates how logistical factors shape final trajectories. Consequently, rates of durable LVAD implantation and spontaneous recovery were lower than those reported in other registries.

Any comparisons between patients managed with Impella 5.5 alone and those requiring combined support should be regarded as descriptive and exploratory, as they are subject to substantial confounding by indication and baseline severity, particularly given the predominance of INTERMACS 1 patients in the combined-support group. Therefore, these findings should not be interpreted as evidence of superiority of one support strategy over another, but rather as reflecting differences in clinical presentation and initial haemodynamic severity.

### Comparison with international evidence

When outcomes are analysed according to INTERMACS severity, mortality rates within each category fall within the range reported in international registries.^6–8^^,^^13^ Approximately 60% mortality in INTERMACS 1 patients and 35%-40% in INTERMACS 2 patients are consistent with data from the Cleveland Clinic, the Cardiogenic Shock Working Group, and other contemporary series.^3^^,^^5^^,^^8^ However, no formal adjusted comparison was performed, and no causal attribution can be made between device strategy, baseline severity, centre organization, and the outcomes observed.

In the absence of central adjudication, harmonized complication definitions across centres, a comparator group, or external benchmarking, reported device-related complications should be interpreted as observed event frequencies within this cohort rather than as formal safety benchmarks or evidence of device safety. Although adverse events in our cohort were not centrally adjudicated, the observed frequencies are within the range reported in recent Impella 5.5 series. For example, the multicentre SURPASS registry^6^ reported stroke in 3.6%, renal replacement therapy in 10.4%, bleeding in 27.0%, and clinically significant haemolysis in 13.5%, whereas a smaller single-centre series of prolonged support^3^ described higher rates of bleeding, haemolysis, AKI, and vascular complications, likely reflecting differences in case severity, support duration, and event definitions. Therefore, our complication data should be interpreted as descriptive frequencies rather than as direct safety benchmarks.

INTERMACS stratification should therefore be interpreted as a descriptor of baseline severity, and any considerations regarding timing or optimization of support as hypothesis-generating.

Support duration in the Spanish cohort (mean 16 days) lies between that reported in large U.S. registries and shorter European experiences.^6^^,^^7^^,^^14–17^

### Alignment with contemporary consensus documents

Recent international consensus documents describe structured shock teams, rational device escalation strategies (including ECMO–Impella combinations), and early definition of clinical trajectories.^11^^,^^12^^,^^18–20^ The management patterns observed in this cohort are consistent with these principles, reflecting contemporary practice in tMCS rather than validating specific outcome recommendations.

### Implications for national registries

Current Spanish transplant registry data group heterogeneous support modalities under the category of “mechanical circulatory support,” without differentiating between microaxial pumps or specific Impella models.^10^ This lack of granularity limits assessment of the impact of Impella 5.5 on pre- and post-transplant outcomes. Given its ability to deliver high-flow support and enable mobilization, Impella 5.5 warrants specific identification in future national registries.

Emerging evidence also describes its use in rehabilitation and structured escalation strategies, reinforcing its relevance within contemporary tMCS algorithms.^21^^,^^22^ Similar heterogeneity in support strategies and the influence of baseline severity on outcomes have been reported in other observational series evaluating surgically implanted Impella devices and bridge-to-transplant pathways in cardiogenic shock.^23^^,^^24^

### Limitations

This study has the inherent limitations of a retrospective and observational design, including a degree of heterogeneity among participating centres and the absence of a control group. Complications were reported according to local clinical practice at each participating centre, without central adjudication or formal harmonization of definitions across centres. The absence of systematic data on baseline left and right ventricular function, invasive haemodynamics, vasoactive support intensity, and detailed echocardiographic parameters limits mechanistic interpretation and precludes causal attribution regarding device selection or timing. In addition, pre-shock heart transplant candidacy and potential changes in candidacy during Impella 5.5 support were also not systematically captured across centres, precluding reliable analysis of whether temporary support modified transplant eligibility. Vascular complications and aortic regurgitation prevalence were also not systematically captured across centres, precluding reliable analysis of these variables.

Accordingly, this study is descriptive in nature and focuses on clinical trajectories and organizational patterns rather than mechanistic determinants of Impella implantation. Causal attribution between device-related effects, system organization, and baseline clinical severity is therefore not possible within this study. Extension of survival assessment beyond hospital discharge would have provided a broader prognostic perspective. In addition, all participating hospitals were active heart transplant centres, and these findings may therefore reflect organizational pathways, escalation strategies, and patient selection specific to transplant-oriented institutions rather than to non-transplant hospitals.

Furthermore, although multicentric in nature, the sample size remains limited when compared with large international registries,^6^^,^^8^ restricting statistical power for complex multivariable analyses. The absence of statistically significant differences in some analyses should therefore be interpreted as no differences detected rather than evidence of no effect, particularly in the context of limited sample size and residual confounding.

Nevertheless, this cohort represents the largest experience documented in Spain and the first national multicentre series specifically dedicated to Impella 5.5, providing information that is directly applicable to clinical practice and complementary to data from larger international registries.

## CONCLUSION

Impella 5.5 was used in patients with severe cardiogenic shock, providing more than 2 weeks of support, and was frequently followed by heart transplantation; durable LVAD implantation was uncommon.

Observed device-related complications are reported as event frequencies, and any comparison with published international series should be interpreted as descriptive only. A clear mortality gradient according to INTERMACS profile was confirmed, consistent with the established association between baseline severity and outcomes.

The high proportion of heart transplantation and the increasing use of controlled DCD observed in this cohort should be interpreted within the context of Spanish heart transplant-oriented centres and healthcare systems characterized by high organ availability.

These findings support dedicated registries for short-term mechanical circulatory support that specifically distinguish Impella 5.5, allowing high-resolution data, optimization of patient selection, and better contextualization of its role in cardiogenic shock management and heart transplant pathways across healthcare systems.

## Data Availability

The data underlying this article are available in the article and its supplementary material.

## ABBREVIATIONS

AKI: Acute kidney injury
CA: Cardiac arrest
CrCl: Creatinine clearance
DCD: Donation after circulatory death
DM: Diabetes mellitus
ECLS: Extracorporeal life support
INTERMACS: Interagency Registry for Mechanically Assisted Circulatory Support
KDIGO: Kidney Disease: Improving Global Outcomes
LMCA: Left main coronary artery
LVAD: Left ventricular assist device
MAP: Mean arterial pressure
MI: Myocardial infarction
OTI: Orotracheal intubation
PCI: Percutaneous coronary intervention
RVAD: Right ventricular assist device
SBP: Systolic blood pressure
tMCS: Temporary mechanical circulatory support
VA-ECLS: Venoarterial extracorporeal life support
VV-ECLS: Venovenous extracorporeal life support
